# An assessment of a GMP schistosomiasis vaccine (SchistoShield^®^)

**DOI:** 10.3389/fitd.2024.1404943

**Published:** 2024-07-29

**Authors:** Jiho Kim, Jenn Davis, Jinhee Lee, Sang-Nae Cho, Kiyoung Yang, Jaekyoon Yang, Sungmin Bae, Joohee Son, Boyoung Kim, Dale Whittington, Afzal A. Siddiqui, Darrick Carter, Sean A. Gray

**Affiliations:** 1 PAI Life Sciences, Seattle, WA, United States,; 2 Quratis Corp, Cheongju, Republic of Korea,; 3 Department of Microbiology, Yonsei University, Seoul, Republic of Korea,; 4 Department of Medicinal Chemistry, University of Washington, Seattle, WA, United States,; 5 Department of Immunology & Molecular Microbiology, Center for Tropical Medicine & Infectious Diseases, Texas Tech University Health Sciences Center, Lubbock, TX, United States,; 6 Department of Global Health, University of Washington, Seattle, WA, United States

**Keywords:** schistosomiasis vaccine, quality assessment, vaccine development, technology transfer, vaccine trials, Sm-p80, SchistoShield ^®^

## Abstract

**Introduction:**

Schistosomiasis is a neglected tropical disease that puts over 200 million people at risk, and prevention options are sparse with no approved vaccine. Our vaccine candidate, SchistoShield^®^, is based on an approximately 87 kDa large subunit of calcium activated neutral protease - termed Sm-p80 - combined with a potent TLR4 agonist-based adjuvant. SchistoShield^®^ has been shown to prevent disease throughout the parasitic life cycle - including egg, juvenile, and adult worm stages - in numerous animal models up to and including baboons. SchistoShield^®^ has been shown safe in both preclinical toxicology studies in rabbits and in a Phase 1 clinical trial in the USA. A Phase 1b trial was initiated in 2023 in endemic regions of Africa, and to date no serious safety signals have been reported.

**Methods:**

In preparation for large-scale Phase 2 clinical trials and eventual vaccine deployment, the Sm-p80 antigen production process has been transferred to a manufacturing organization, Quratis Corporation in South Korea, which specializes in preparation of vaccines for large-scale European and African trials. The process of scaling from our current production level of ~2000 vaccine doses, to a process that will generate more than 100 million doses has required multiple improvement steps in the process including fermentation, downstream purification of the protein antigen, lyophilization, and fill and finish.

**Results:**

In this study, we detail the large-scale production process of the SchistoShield^®^ protein product by Quratis. In addition, an effort was made to analyze and compare the Quratis-made lot of Sm-p80, referred to as QTP-105, to the cGMP lot of Sm-p80 which is in use in human trials in the USA and Africa, referred to as Sm-p80 DP (made in USA). We show that QTP-105 demonstrates excellent potency, purity, identity, and endotoxin levels compared to our Phase 1 Sm-p80 DP and is suitable for use in Phase 2 studies and beyond.

## Introduction

Schistosomiasis is a neglected tropical disease caused by multiple species of the genus, *Schistosoma*, a type of blood fluke (1). Depending on the species of *Schistosoma*, the disease can manifest as intestinal schistosomiasis (S. *mansoni, S. japonicum, S. mekongi*.) or urogenital schistosomiasis (*S. hematobium*). Manifestation and symptoms are diverse, with symptoms ranging from abdominal pain, fluid accumulation in the peritoneal cavity, and liver enlargement, all of which can contribute to significant morbidity and death (2). The disease affects approximately 200 million people annually, affecting populations mostly in the tropics (*i.e*. Sub-Saharan Africa and Southeastern Asia). Treatment is available in the form of the anti-parasitic drug praziquantel, and is effective in reversing the symptoms of the disease with an annual dose of treatment (3). However, the drug itself is ineffective against immature parasites, does not prevent extremely common reinfections after initial elimination, and the costs of treatment are restrictive in the most endemic environments (4).

With the limitations of praziquantel, a vaccine against schistosomiasis remains a highly desirable option for schistosomiasis control and prevention. However, no vaccines have been approved for human use as of present - although a few efforts are ongoing (5–7). Our candidate vaccine, SchistoShield^®^, is comprised of the recombinant Sm-p80 antigen produced in *E. coli* combined with the adjuvant glucopyranosyl lipid adjuvant in a stable oil and water emulsion (GLA-SE). Sm-p80 is so named as it is the large 80 kDa subunit of the antigenic calcium-dependent neutral protease (calpain) expressed by *Schistosoma mansoni*. This antigen is a surface-associated amphitropic protein found in nearly every parasitic life stage making it an ideal vaccine target (8). In *Schistosoma* worms, Sm-p80 helps the parasite to evade immune responses through its surface membrane renewal process continually evading the immune response by remodeling the parasite membrane (9, 10). The adjuvant used in combination with Sm-p80, GLA-SE, is an oil-in-water emulsion containing a toll-like receptor 4 (TLR4) agonist (GLA) formulated in a squalene nanoemulsion. Use of GLA-SE as an immunological adjuvant has been extensively investigated in combination with antigens against infectious diseases such as tuberculosis, influenza and leishmaniasis (11–13). The antigen-adjuvant combination has seen high effectiveness in the prevention of schistosome transmission by significant reduction of egg-producing female parasites in baboons (14). Numerous studies have verified the efficacy of SchistoShield^®^ at providing protection in extensive preclinical studies involving mice and baboons emulating schistosome infection (5, 15). Vaccination with SchistoShield^®^ results in strong protection against not just against the homologous pathogen, *S. mansoni*, but also against the related parasite *S. haematobium* which causes urinary schistosomiasis.

We have recently completed GMP manufacture, fill-finish, lyophilization, and release of the Sm-p80 antigen for use in its first ever human phase 1 clinical trial against schistosomiasis (16). This phase 1 dose-ranging trial in non-endemic settings was started in May of 2022 and follow up will be completed in June of 2024. As of the writing of this manuscript, there have been no serious safety signals generated in the low, mid, and high-range doses even after 4 doses. In November of 2023, we initiated a follow-on phase 1b trial in schistosomiasis endemic regions of Africa, specifically Madagascar and Burkina Faso.

Initial process development and optimization was carried out at PAI Life Sciences. For Phase 1 and 1B trials, Sm-p80 was manufactured under GMPs at the University of Iowa Center for Biocatalysis and Bioprocessing (CBB) (Coralville, IA), and filled, lyophilized, and finished at Lyophilization Technologies, Inc. (Warminster, PA). Multiple replicative pre-GMP engineering runs produced 3 reference lots of Sm-p80, one of which was used in GLP toxicology studies in rabbits. GMP production runs generated approximately 2000 doses of Sm-p80 DP which was used for the US Phase 1 trial, and the African Phase 1b trials. As we prepare for Phase 2 trials, there was a regulatory requirement for a manufacturer who can produce GMP phase 2 products that are compliant with EU and African requirements and has the facilities and infrastructure to produce millions to billions of doses. For this we chose the Contract Manufacturing Organization (CMO) Quratis Corporation, located outside of Seoul, South Korea. PAI Life Sciences worked closely with Quratis to transfer processes for fermentation, inclusion body (IB) recovery, purification, and lyophilization scaling the process up nearly 100-fold. In this paper, we outline the process of verifying the drug product (DP) and drug substance (DS) manufactured by Quratis Corp. to confirm product quality and potency in comparison to the initial GMP protein production runs. The drug product passed all of the testing criteria and demonstrated potency in eliciting anti-Sm-p80 IgG antibodies in mice. This represents a significant step in verifying clinical safety and efficacy of the investigational product in preparation for clinical trials to be held later in 2024.

## Materials and methods

### Protein and adjuvant production at PAI Life Sciences and AAHI

The adjuvant GLA-SE was manufactured by and purchased from the Advanced Access to Health Initiative (AAHI) following a manufacture process published previously (17). Sm-p80 antigen was manufactured at Quratis Corp. (QTP-105) and at University of Iowa CBB (Sm-p80 DP) according to earlier published protocols in a prokaryotic production system (18). In brief, the coding sequence of Sm-p80 (GenBank Accession Number M74233) was cloned into a pET29a-based vector using a gap repair cloning strategy, which was subsequently transformed into a HMS174(DE3) *E. coli* strain. A non-GMP Research Cell Bank (RCB) of *E. coli* strain expressing Sm-p80 was produced and optimized by PAI Life Sciences Inc. and used to develop the original Sm-p80 process. GMP production at CBB involved a fermentation process at a 15 L scale followed by a purification process targeting the Sm-p80 protein. Briefly, the prepared 30-L fermenter containing 15-L of growth media was inoculated with the Sm-p80 starter culture and was allowed to reach an OD600 of 4.0 before induction with IPTG. The fermenter was maintained at pH 7.0, 37°C and DO set point of 40% (DO was controlled by agitation at 400 – 700 rpm and aeration at 30 lpm followed by Oxygen supplementation as needed). The fermenter was harvested 3 hours post induction by centrifugation and the biomass was weighed and stored as one aliquot at −80°C. The purification process for the GMP run at CBB was at a 25g IB scale. The IB was isolated then solubilized prior to purification through a 3-column process using 2 ion exchange resins and a hydrophobic interaction resin. The scale purification process developed for Sm-p80 antigen involved purification through a 500 mL Capto-S column, followed by a 500 mL Capto-MMC, and 500 mL Capto-butyl columns (Cytiva, Marlborough, MA). This process was used to purify the final product for subsequent use in phase 1 and 2 clinical trials. The Quratis process transfer development was performed at a 2 – 5L fermentation scale followed by a purification scale at 20 – 100 mL of resin. At the time of comparison, the University of Iowa CBB product had been stored at 4°C for 24 months prior to the potency testing comparisons with the fresh QTP-105 product.

### Sm-p80 protein (QTP-105) manufacture and lyophilization at Quratis Corp

The Sm-p80 RCB described above was tested at Charles River Laboratories (CRL, Malvern, PA) and a Certificate of Analysis (CoA) issued ensuring suitability for downstream cell banking. A GMP Master Cell Bank (MCB) and Working Cell Bank (WCB) were produced from the RCB at Charles River Laboratories. Both the MCB and WCB were thoroughly tested and released by CRL and CoAs issued ensuring suitability for GMP production. Initially, there was a technology transfer from PAI Life Sciences to Quratis regarding a small scale (5 L fermentation) Sm-p80 manufacturing. Upon successful completion of small-scale production, scale-up to the 500 L fermentation level was carried out for commercialization and wider distribution.

The fermentation of *E. coli* expressing Sm-p80 was performed in a 500 L fermenter (Biozeen, India) at Quratis’ GMP facility. The culture medium used for seed cultures contained soy peptone 12 g/L, yeast extract 24 g/L, and glycerol 5 g/L with an addition of 20× potassium phosphate media (K_2_HPO_4_ 250.8 g/L, KH_2_PO_4_ 46.2 g/L, MgSO_4_*7H_2_0 3 g/L) to the medium to adjust the pH to 7. The main culture medium was identical to the seed cultures except that the main culture media contained 10% glycerol, as compared to 5% glycerol for the seed cultures. The culture media were autoclaved before use. The culture volumes were escalated gradually through two rounds of seed cultures up to a 300 L main culture. The volumes of the first and second seed cultures were 1.2 L and 30 L, respectively. The second culture that had been grown to >12 AU (absorption unit) at OD600 nm was transferred to the main culture to constitute 10% of the main culture. The temperature for both seed cultures and the main culture were maintained at 37°C. The culture conditions for the main culture (300 L) were 300 rpm agitation speed, 300 L/min aeration rate, and the pH was maintained at 7.0 by adding acid and base buffers (6N HCl and 6N NaOH). When the main culture reached >5 AU at OD600nm, IPTG was added to a final concentration of 1 mM IPTG in the culture to induce Sm-p80 expression. The culture was terminated 6 hours of the start of IPTG induction when dissolved oxygen levels were observed to steeply elevate. The final density of the main culture was around 50 AU at OD600nm.

After fermentation, the culture was transferred to a continuous centrifuge to harvest the bacteria as a slurry and stored at −65 °C until use. After the slurry was reconstituted in resuspension buffer (20 mM Tris, pH 8.0), the bacteria were subjected to three disruption cycles using a Microfluidizer (PICOMAX(MN400F), Micronox, South Korea) at 18,000 psi. The resulting solution was centrifuged to collect inclusion bodies (IB), and the IB were washed three times using wash buffer (20 mM Tris, pH 8.0). The final IB pellets were then dissolved in lysis buffer (20 mM sodium phosphate, 50 mM sodium chloride pH 8.0) containing 8M urea for 24 h. Lysates were centrifuged to remove any precipitate and the supernatants were filtered through a 0.45 μm filter. The IB lysate containing the Sm-p80 protein was subjected to three steps of purification, including Q Sepharose FF (Cytiva, Marlborough, MA), Butyl Sepharose ImpRes (Cytiva), and Capto MMC (Cytiva) resin columns. As the first step, the lysates were loaded onto a Q Sepharose FF resin column - a strong anion exchange chromatography matrix – and the Sm-p80 protein allowed to bind to the resin. The column was washed with washing buffer (20 mM Tris, 8 M urea, and 50 mM sodium chloride pH 8.0) and was eluted with elusion buffer (20 mM Tris, 8 M urea, and 1300 mM sodium chloride pH 8.0). The elutes were adjusted to a conductivity of 75 mS/cm using ammonium sulfate and loaded onto a Butyl Sepharose ImpRes resin column - a hydrophobic interaction matrix - for the second purification step. The elutes from the first purification were first equilibrated in equilibration buffer (20mM Tris, 10% glycerol, 0.6 M ammonium sulfate, 8M urea, pH 8.0) and loaded onto the column. The column was washed with washing buffer (20mM Tris, 10% glycerol, 0.6M ammonium sulfate, 8M urea, pH 8.0) and eluted with elution buffer (20mM Tris, 8M urea, pH 8.0). Lastly, the Sm-p80 protein was polished using a Capto MMC resin column, a multimodal chromatography. The elutes from the second purification step were loaded onto the Capto MMC column and washed with washing buffer (20mM Tris, 140mM sodium chloride, 8.0M urea pH 8.0) and eluted with elution buffer (20mM Tris, 240mM Sodium Chloride, 8.0M Urea pH 8.0). The third elutes were buffer changed into formulation buffer (20 mM Tris, pH 8.0) using a 10 kDa ultrafiltration (UF) membrane (Repligen USA), until more than 1000 volumes of buffer change occurred. The resulting protein concentration was 1.0 mg/ml as measured by absorbance at 280nm, and this Sm-p80 solution (drug substance, DS) was filtered through a 0.22 μm filter for sterilization and stored in a polycarbonate container at −65 °C.

The Sm-p80 drug product (DP, QTP-105) was manufactured by adding mannitol, sucrose, polysorbate 80 as excipients, and - after filter sterilization - DS was dispensed into 3 ml vials at an amount of 125 ug of Sm-p80 protein each. The liquid in the vials were lyophilized (L.M-10, CnH, Korea) and stored at 2–8°C. The lyophilization cycle comprised pre-freezing and drying steps where temperature, time, and vacuum pressure had been optimized for DP manufacturing. The water content measured by the Karl Fischer method was less than 2.5%.

### Testing and verification processes

Vials containing lyophilized protein were resuspended in sterile water for injection (WFI) to a final concentration of 0.2 mg/mL. Physical characteristics were observed immediately prior to and subsequent to the resuspension process. Successful resuspension was defined as absence of any visible particulate matter in the vial within 30 seconds of addition of water to the vial, with the final resuspended product being a colorless liquid. Protein concentration was re-verified by absorbance at 280nm and confirmed by running the protein at a 1 μg load on a 4–20% glycine gel electrophoresis after boiling (98°C for 10 minutes) and reduction using dithiothreitol (DTT). A sodium dodecyl sulfate (SDS)-containing running buffer was used. The gel was subsequently washed with warm water, followed by SimplyBlue staining (ThermoFisher Scientific) for 1 hour. Gels were destained in deionized water for 1 hour. The intensity of the protein bands were quantified using ImageJ densitometry analysis by comparison with a commercially available standard loaded mass (1μg, ThermoFisher, Waltham, MA, USA) of bovine serum albumin (BSA) in triplicate as a comparator (19).

Western blots were performed by transferring a gel after electrophoresis using an iBlot2 (ThermoFisher Scientific) and blocked in 2% non-fat dairy milk in phosphate buffer saline with tween (PBS-T). Primary antibodies were then diluted according to standard concentrations. Baboon anti-Sm-p80 serum (Texas Tech University, Lubbock, USA) was used at a 1:3000 dilution followed by a 1:40000 dilution of secondary Goat anti-monkey IgG(H+L)-HRP (Thermo-Scientific, Waltham, USA). Monoclonal anti-Sm-p80 Smab14 (PAI Life Sciences, Seattle, USA) was used at a 1:64000 dilution followed by a secondary 1:5000 dilution of Goat anti-mouse IgG-HRP (Southern Biotech, Birmingham, USA). For detection of *E. coli* host cell protein, a 1:1000 dilution of anti-*E. coli* HCP antibody (Rockland Immunochemicals Inc., Pottstown, USA) was followed by a 1:2000 dilution of secondary donkey anti-rabbit IgG(H+L)-HRP (Southern Biotech, Birmingham, USA). Membranes were incubated with primary antibody dilutions for 1 hour at room temperature and washed 3× with PBS-T. Secondary antibodies were diluted according to the stated concentrations, and washed 3× with PBS-T. Membranes were developed using Ultra-TMB ELISA (ThermoFisher Scientific, Waltham, MA, USA) and left to develop for 10 minutes before terminating by submerging the gel in water. Residual *E. coli* host cell DNA quantification was carried out at locations adhering to US FDA Good Manufacturing Practice Regulations (21 CFR 210, 211). In short, DNA was extracted from the product and quantified for *E. coli* DNA using quantitative polymerase chain reaction (qPCR).

Mass spectrometric and peptide mapping analyses were done on QTP-105 and GMP DP. 50 uL of QTP-105 and Sm-p80 DP resuspended product (equivalent to 10μg total mass) were taken for trypsin digest. Proteins were digested using a Promega rapid digestion trypsin kit (Promega Corp, Madison, WI, USA). Proteins were first reduced and alkylated using tris(2-carboxyethyl)phosphine (TCEP) and iodoacetic acid respectively. Digest was carried out according to manufacturer’s kit reagents and protocols.

Digested products were run on LC-MS on a an Orbitrap Ascend mass spectrometer (ThermoFisher Scientific, Waltham, MA, USA). Liquid chromatography was performed using a Waters nanoEase M/Z Peptide CSH C18 130Å, 1.7μm 75μm × 250 mm column (Waters, Milford, MA, USA). A gradient of Solution A (0.1% Formic acid in water) and B (0.1% Formic acid in acetonitrile) was used, starting at a ratio of 95:5 solution A:B, ramping gradually up to 15:85 A:B at 122 minutes after start, before re-equilibration at 95:5 A:B at 145 minutes after start. Ionization source was nanospray (NSI), with a scan range of 400–2000 m/z with an Orbitrap detector.

Raw data were run through BioPharma Finder (Thermo Fisher Scientific, Waltham, MA), with peptide analysis done on the Sm-p80 protein sequence. An *E. coli* host cell protein library consisting of 4404 unique proteins was downloaded from Uniprot (UP000000625) for host cell protein contaminant detection. A comparator Chinese hamster HCP library was by default integrated into the software for HCP analysis.

### Potency assay of Sm-p80 DP and QTP-105

Sm-p80 proteins were resuspended in deionized water to a concentration of 0.2 mg/mL. C57/BL6 mice (n=10 per group) were given an individual dose of 10 μg of antigen (protein) and 1 μg of adjuvant (GLA-SE). Antigen and adjuvant were kept in sterile conditions and mixed immediately prior to injection into mice. Mice were injected intramuscularly with 100 μL of antigen/adjuvant mixture, with 50 μL being injected into each hind leg. Serum samples were isolated from each mouse prior to immunization and on day 14 after injection. Approximately 100 μL of blood were collected in Multivette^™^ Serum Gel tubes (Sarstedt AG, Nümbrecht, Germany). Samples were kept at room temperature for at least 15 minutes before being centrifuged at 10000 RPM for 10 minutes and the upper serum layer was extracted for storage. The serum samples were stored at −80°C until subsequent use. Serum samples were tested for anti-Sm-p80 IgG antibodies by ELISA. In short, Corning high binding 384-well plates (Corning, NY|) were coated with Sm-p80 protein (PAI Life Sciences, Seattle WA) in carbonate buffer. The coated plates were blocked prior to the addition of diluted serum samples. The mouse anti-Sm-p80 activity was detected using a HRP label Goat anti-Mouse IgG (H+L) polyclonal antibody (Southern Biotech, Birmingham, AL) and visualized with acidified TMB (SeraCare, Milford, MA). The reaction was read out at 450nm, and activity was analyzed using XLFit (IDBS, Woking, United Kingdom) analysis. Samples were considered positive if the mean OD450 of each sample was greater than the mean + 6 standard deviations (SD) of the pre-immunization sera. The acceptance criteria for seroconversion was greater than 80% of mice becoming positive as defined, 14 days following immunization. This criteria is in line with what was accepted by the US Food and Drug Administration (FDA) for the evaluation of the Sm-p80 DP in the 5-year stability program.

## Results

### Physical appearance of GMP-compliant Sm-p80 drug product

**FIGURE 1:**
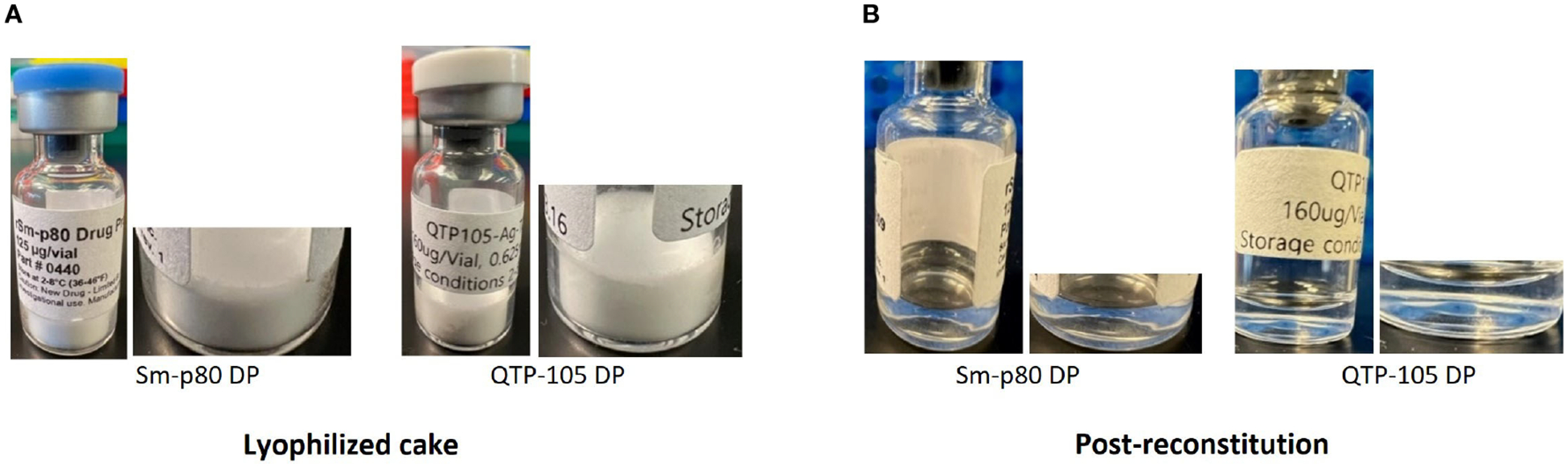
Physical appearance of Sm-p80 DP and QTP-105 drug product (DP) before **(A)** and after **(B)** reconstitution in sterile water for injection (WFI). Post-reconstitution photos were taken within 5 minutes after complete reconstitution.

Sm-p80 protein was manufactured and vialed at a 5 L scale at the Quratis facility under the trade name QTP-105. This was part of ongoing efforts to advance the Sm-p80 based schistosomiasis vaccine candidate to a higher-scale manufacturing for worldwide distribution. The process development at Quratis evolved to accommodate scaled up manufacturing and improve the quality of the Sm-p80 protein from previously established protocols, namely by using a pET29a-vector based construct in HMS174 *E. coli* under fermentation conditions developed by PAI Life Sciences Inc. in Seattle, WA. Seven vials each of the lyophilized drug product (DP) and bulk drug substance (BDS) were delivered to PAI Life Sciences Inc. for analysis and comparison to previous SchistoShield^®^ GMP DP manufactured at University of Iowa CBB, which had reached their 24-month stability time point. (Table 1). Physical verification of the products indicated an excellent white cake as expected after successful lyophilization, with no presence of any unexpected particulates or moisture. Upon resuspension of product in deionized water, the process took less than 30 seconds, and the appearance of the product was a clear liquid without any particulate or precipitate present, as expected (Figure 1 and Table 2A).

### Protein characterization of Sm-p80 drug product – quantity and identity

**FIGURE 2:**
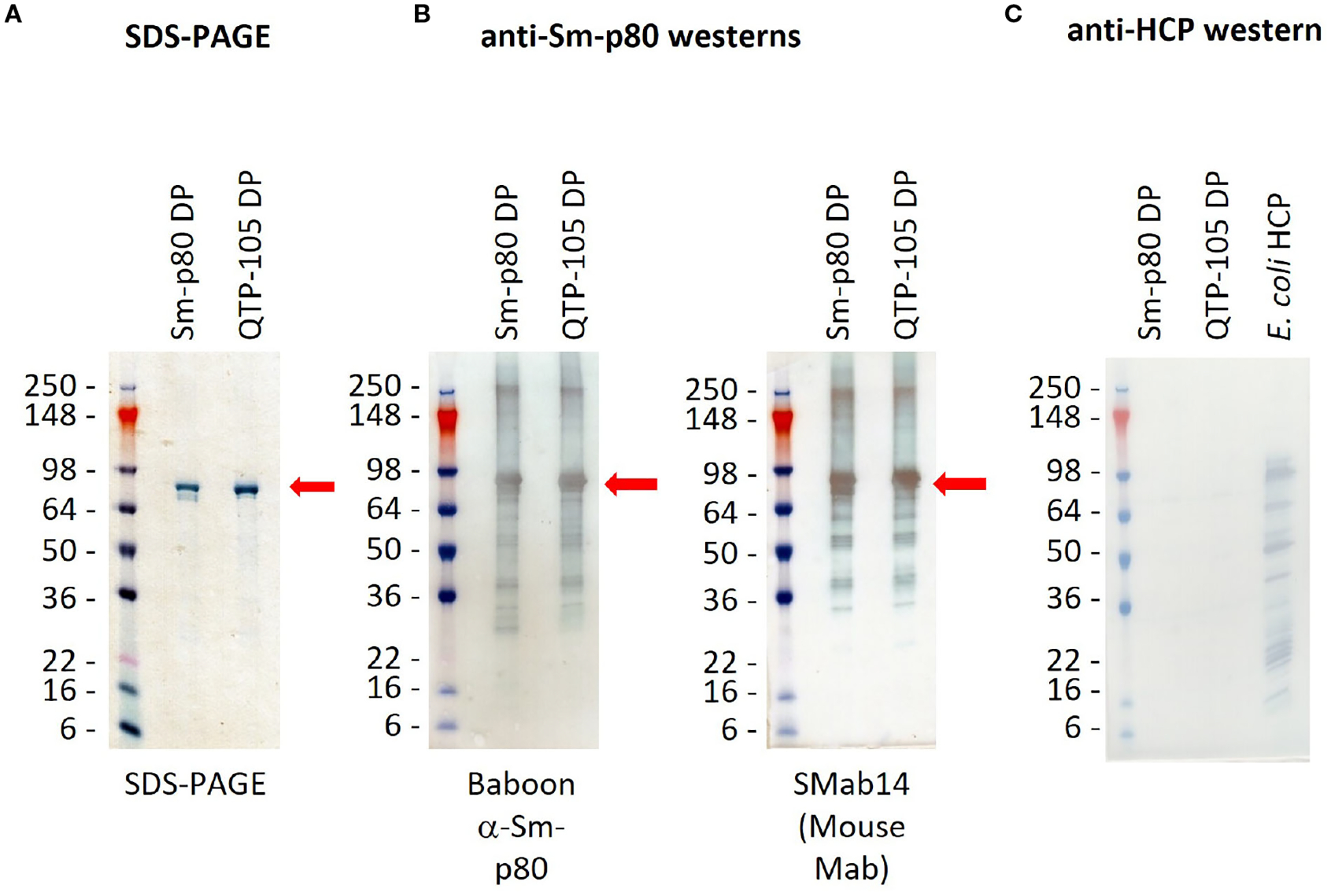
SDS-PAGE gel **(A)** and western blots **(B, C)** confirming identity and purity of Sm-p80 protein in Sm-p80 DP and QTP-105. Gels were transferred onto membranes before incubation with primary and secondary antibody dilutions according to methods. Sm-p80 protein was identified by use of immunized baboon sera and a monoclonal mouse antibody (SMab14) **(B)**, and absence of E. coli host cell proteins was verified for purity **(C)**. Sm-p80 bands are indicated with red arrows. Pictures were taken within 5 minutes of termination of TMB-HRP reaction.

Resuspended Sm-p80 DP and QTP-105 were run on a sodium-dodecyl sulfate-polyacrylamide gel electrophoresis (SDS-PAGE) for quantification and purity analysis. The QTP-105 product showed a similar product purity profile when compared to the GMP DP product, with a prominent protein band at approximately 80 kDa, which is in line with the theoretical mass of the Sm-p80 protein at 87 kDa (Figure 2). Notably, smearing below the prominent band, which indicates the presence of degradation products, was diminished in QTP-105, indicating higher purity and quality compared to the GMP DP. Quantification of protein and protein purity was carried out by comparing the prominent protein band to a standard load mass of BSA. QTP-105 indicated an average purity of 93.3% and the GMP product 78.5%, confirming the superior purity of the QTP-105 (Table 2B).

To further confirm the identity of the protein, a series of Western blots were carried out on the products. Two Sm-p80 specific antibodies were used, one sourced from pre-immunized baboon sera and a mouse monoclonal antibody (Smab14). The Western blots indicated presence of Sm-p80 in both the GMP DP and QTP-105, indicating that the degradation products also contained Sm-p80 antigenic sites (Figures 2A, B). Residual *E. coli* DNA was quantified at 0.01 pg/μg and 1.65 pg/μg for GMP DP and QTP-105 respectively, which were well below the accepted limit. An anti-host-cell protein (HCP) western blot indicated no presence of *E. coli* sourced bacterial contaminants (Figure 2C), further confirming the quality and identity of the proteins.

**FIGURE 3:**
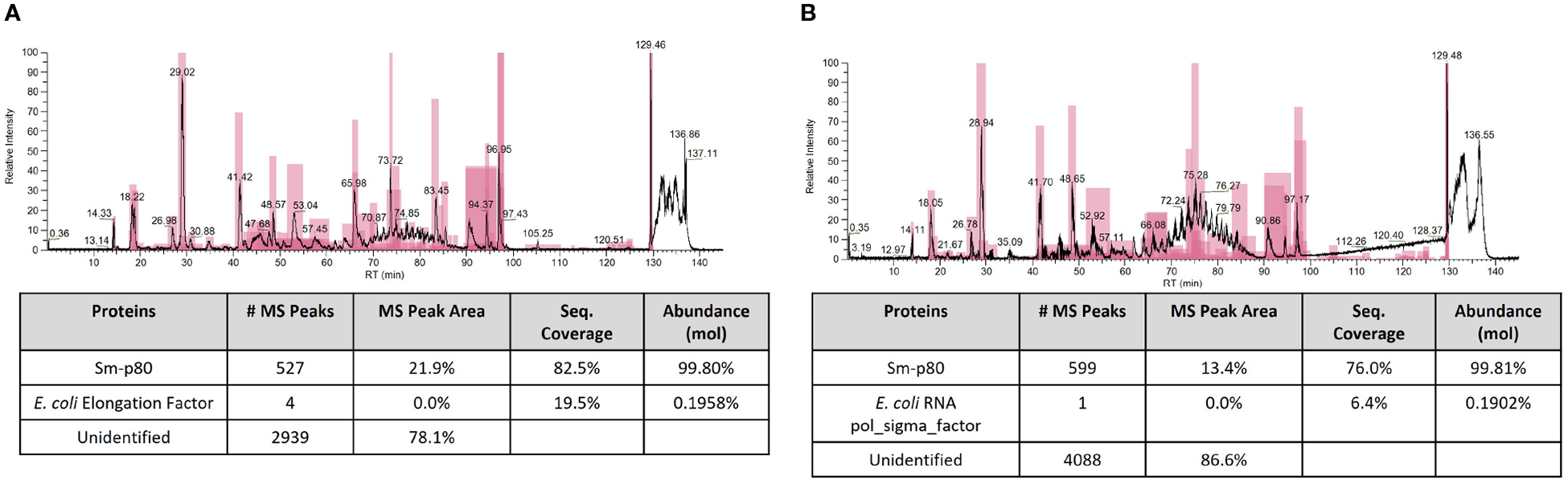
Mass spectrometric (MS) peptide mapping analyses of Sm-p80 DP **(A)** and QTP-105 **(B)**. Products were digested with trypsin before running on stated columns. Resulting peptide products were run against theoretical Sm-p80 digest products, and against the theoretical *E. coli* host cell protein digest products using ThermoFisher BiopharmaFinder software. Data shown includes the MS chromatogram and the peptide mapping results, with total hits against Sm-p80 protein and *E. coli* HCP.

In order to further confirm identity of Sm-p80 protein and presence of any host cell protein (*E. coli*) contaminants, QTP-105 and Sm-p80 GMP DP were digested with trypsin before analysis with mass spectrometry. Peptide mapping analysis indicated a protein coverage of 82.5% and 76.0% for QTP-105 and Sm-p80 DP respectively, a satisfactory coverage for large protein digests (Figure 3). Additionally, the digested products were also mapped against the E. coli host cell protein library to detect presence of any contaminants from the manufacturing process. QTP-105 digest indicated a match with only 4 peptides originating from 1 *E. coli* protein with a relative abundance of 0.0% by peak area, indicating a negligible amount of contaminant. Sm-p80 GMP DP digest indicated a match of 1 peptide from 1 *E. coli* protein with a relative abundance of 0.0% by peak area, again indicating no contamination (Figure 3). A peptide mapping analysis of products against a Chinese hamster host cell protein library, an irrelevant HCP library, indicated similar numbers of hits against peptides (data not shown), thus indicating that the matches against *E. coli* library may be an artifact. The mass spectrometric analysis confirmed the matching identity of the two products against Sm-p80 protein, and indicated minimal presence of host cell protein contaminants.

### Stability and potency of Sm-p80 drug product

**FIGURE 4:**
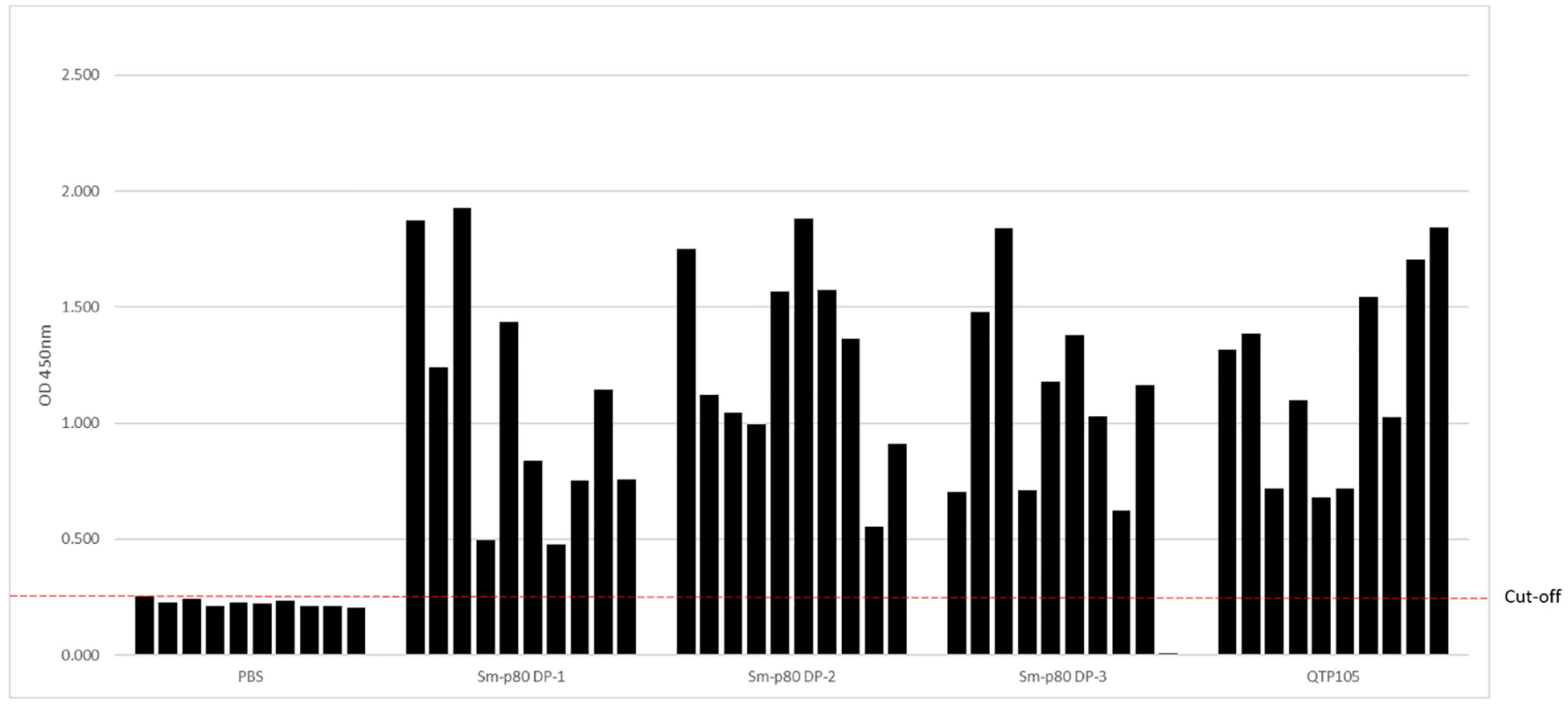
Potency of Sm-p80 DP and QTP-105 products in mice. Mice (n=10 per group) were immunized using 3 different vials of Sm-p80 DP and 1 of QTP-105. Day 14 after immunization, serum samples were taken for analysis of anti-Sm-p80 IgG by ELISA. Cutoff for negatives is shown as a red dotted line.

The QTP-105 or the GMP DP Sm-p80 was administered by a single intramuscular injection in mice to examine whether a satisfactory antibody response is raised against the antigen. Ten micrograms of DP protein were mixed with 1 μg of GLA-SE in a bolus of 0.1 mL prior to injection into C57/BL6 mice. A total of 3 groups, each with 10 mice per group, were injected with Sm-p80 DP + GLA-SE as part of an ongoing potency assay designed to monitor the life of the DP (24-month timepoint). One group of 10 mice was injected with QTP-105 DP + GLA-SE, and the final group of 10 mice received 1 μg of GLA-SE only as a control. Serum was collected at pre-injection (D0) and at day 14, and post-injection titers were tested for anti-Sm-p80 responses by ELISA. By day 14 post-injection, all 10/10 mice passed the cut-off OD in the QTP-105 + GLA-SE group, and all 3 vials of the GMP DP + GLA-SE also passed the minimum requirement of 8/10 mice (Figure 4), with a total of 28/29 mice passing the criteria for potency in mice, across three different vials of GMP DP product. Mice displayed no significant pathology or symptoms throughout the duration of the experiment. Thus, QTP-105 demonstrates equivalent efficacy in raising anti-Sm-p80 antibodies in comparison to the original GMP product, and both products raise a satisfactory immune response against the original antigen within 14 days.

## Discussion

In this series of experiments, we demonstrated equivalence of a small scale to large scale anti-schistosomiasis protein vaccine candidate, produced in a facility in South Korea for eventual use in phase 2 human clinical trials. The scaled product was compared with multiple tests, *i.e*. physical, biochemical, and potency and found to be equivalent to a previously produced batch of Sm-p80 protein in a GMP-compliant setting. This represents a vital next step in the process of advancing the vaccine candidate to the next stage clinical trial setting, where a stable, potent, and easily mass-produced product is necessary.

QTP-105 showed satisfactory equivalent physical and biochemical conditions when compared to the previously manufactured GMP product. The lyophilization process was satisfactory in that a well-formed white cake was produced in the process with no obvious physical defects. An ideal vaccine product for use in clinical trial would be expected to last up to 5 years as a lyophilized product and be able to withstand shipping to remote, sub-Saharan destinations where schistosomiasis is endemic. Original lots of Sm-p80 DP are now 4 years old and have retained their potency. Similar stability programs are being performed on QTP-105 to verify product reliability and potency for up to 5 years.

Although QTP-105 seems to demonstrate superior quality and purity in comparison to the University of Iowa CBB GMP product, it should be noted that the GMP product had reached its 36-month stability time point, whereas QTP-105 was approximately 10 months old when tested. A parallel testing of both products at the same time points would be ideal, but the staggered timing of manufacture would render this impossible to carry out. The QTP-105 product will be tested accordingly to ensure a favorable stability and potency profile which may allow parallel analyses and comparisons with the GMP DP product.

The SchistoShield^®^ vaccine candidate is currently undergoing phase 1b clinical trials in Madagascar using the Sm-p80 DP product produced at UI-CBB. The phase 2 trial and eventual African deployment will necessitate a much larger number of doses and will be produced by Quratis Corp. The process has been well established and can be scaled up readily to reach the number of doses required for future trials. This initial investigation of their pre-GMP drug product suggests that Quratis has the process, ability to scale as needed, and the facilities to manufacture good-quality, potent, and durable product. Based on these comparative analyses, we will be filing an Investigative New Product (IND) application with the Korean Ministry of Food and Drug Safety (KMFDS) for use of QTP105 in an African Phase 2 clinical trial that may finally result in an effective vaccine against schistosomiasis.

## Figures and Tables

**TABLE 1:** Overview of multi-stage development of Sm-p80 vaccine product.

Phase	Step	Scale	Location
Non-GMP Process Development	Fermentation	2–5 L	PAI Life Sciences (Seattle, USA)
	Chromatographic Purification	0.02 – 0.1L (Resin volume)	
	Lyophilization	1000 doses	
GMP Phase 1 (USA) and 1b (Africa)	Fermentation	15 L	University of Iowa Center for Biocatalysis and Bioanalysis (UI CBB, Iowa City, USA)
	Chromatographic Purification	0.5 L (Resin Volume)	UI CBB
	Lyophilization	2000 doses	Lyophilization Technology Inc. (Warminster, USA)
GMP Phase 2 (Africa) and Vaccine Deployment	Fermentation	50–500 L	Quratis Corp. (Cheongju, South Korea)
	Chromatographic Purification	5–25 L (Resin Volume)	
	Lyophilization	>10 Million doses	

**TABLE 2:** Comparison criteria for Sm-p80 and QTP-105 products.

Table 2A. Comparison criteria for Sm-p80 BDS and QTP-105 BDS.			
Criteria	Sm-p80 BDS	QTP-105 BDS	Pass Criteria
Quality of liquid BDS solution (Observe for particles or precipitation)	No presence of particles or precipitation	No presence of particles or precipitation	No presence of particles or precipitation
Color of solution (should be clear to slightly opalescent)	Clear	Clear	Clear
Table 2B. Comparison criteria for Sm-p80 DP and QTP-105 DP.			
Criteria	Sm-p80 DP	QTP-105 DP	Acceptance Criteria
Quality of lyophilized cake (Observe for air bubbles, pulling away from sides of the vial; collapsing of cake)	No presence of air bubbles; Cake adheres to sides of the vial. No shrinkage of cake	No presence of air bubbles; Cake adheres to sides of the vial. No shrinkage of cake	White to off-white cake, cake adherent to vial without signs of shrinkage
Presence/absence of moisture	No moisture present; 1.4%	No moisture present; 1.4%	None present (defined as <4% w/w)
Reconstitution in dH_2_O after 30 seconds	Complete	Complete	Complete
Presence/absence of particulate matter in resuspended solution	None seen	None seen	No signs of obvious contamination, foreign matter, or damage that could affect product quality.
Concentration	0.23 mg/mL	0.21 mg/mL	0.16 – 0.24 mg/mL
Purity of 80 kDa band by Image J	78.5%	93.3%	One dominant band
Endotoxin	1013 EU/mg	2010 EU/mg	<3000 EU/mg
*E. coli* host cell DNA	0.01 pg/μg	1.65 pg/μg	<100 pg/μg
Potency in mice (#positive/#total)	28/29 (97%)	10/10 (100%)	>80% Seroconversion after 14 days

## Data Availability

The raw data supporting the conclusions of this article will be made available by the authors, without undue reservation.
